# The Herbst Clinics in America

**Published:** 1886-10

**Authors:** 


### THE HERBST CLINICS IN AMERICA.

REPORTED EXPRESSLY FOR THE INDEPENDENT PRACTITIONER.

(Continued from page 519.)

### CLINIC GIVEN ON WEDNESDAY, JULY 7TH, AT THE NEW YORK COLLEGE OF DENTISTRY.

Dr. Herbst filled for Mr. J. R. De Silva, of South America, a student of the above named college, the labial surface cavity of the right upper canine, in six minutes, with Wolrab gold cylinders. The ring clamp was applied to hold the rubber dam above the cavity. He also filled for the same patient the mesial surface of a right upper lateral in ten minutes and a half, using the same gold. For purposes of demonstration he constructed a shellac matrix in the following manner. A piece of shellac about the size of a pigeon's egg was heated over an alcohol lamp to the consistency of putty, and after the rubber dam had been applied it was pressed behind the lingual surface, and a little over the cutting edges of the six front teeth. After removal, pieces of steel spring were heated and inserted in such position that they would rest between the centrals and laterals. The whole of the matrix was then replaced in the mouth, to insure the proper fitting of the steel spring between the approximate surfaces of the lateral and central teeth. When such a matrix is to be used it should be made previous to excavating, that the shellac may not be pressed into the cavity. If, however, the opening is so large that the shellac finds entrance, it may be trimmed away with a cold excavator. Great care should be exercised that the piece of steel which has been pushed between the approximate surfaces of the teeth is free from adhering shellac. If this be not the case, the burnisher may incorporate some of the shellac into the gold, and hence the next layer will not be easily adjusted. For the same reason, the first layer of gold introduced into the cavity should be sufficiently large to cover the shellac entirely.

CLINIC GIVEN JULY 7TH, AT THREE P. M., AT THE DEPOT OF THE S. S. WHITE DENTAL MANUFACTURING COMPANY.

Dr. Herbst filled for Mr. J. R. De Silva, of South America, the mesial surface of the left upper canine, with Wolrab's gold cylinders, in five minutes. He also filled the right upper central incisor, mesial surface, in eleven and a half minutes, and the left upper central incisor, mesial surface, in four and a half minutes, using in all of these operations steel instruments in the dental engine. The matrices used for these fillings were made out of a piece of steel spring about eight or ten inches long, and one quarter of an inch in width, and of the thickness of about No. 30 plate gauge, which was pressed between the teeth. That it might be firmly held in position, he inserted it into a piece of shellac, which had been previously softened over an alcohol flame, and while in a soft condition this was pressed into position against the lingual surface of the incisor teeth, allowing a little of the shellac to extend over the cutting edge.

He also filled for Dr. Bödecker a right upper central incisor, the cavity involving about two-thirds of the entire labial surface of the tooth, the enamel of which had been removed by chemical abrasion. The cavity, which was very shallow, was prepared by cutting a fine groove around the border, while the center was left, as it was found perfectly polished. As the abraded surface was very sensitive, the Herbst obtundent was employed with success. The introduction of the gold required about twenty-three minutes. The attendence at this clinic numbered about seventy.

CLINIC GIVEN THURSDAY, JULY 8TH, 8.30, AT THE OFFICE OF DR. WM. CARR.

There were present Drs. Wm. Carr, J. C. Neisley, C. E. Francis, J. R. De Silva, R. Schreiter and C. F. W. Bödecker. Dr. Herbst filled for a private patient of Dr. Carr the right lower second bicuspid, the cavity occupying the distal mesial and grinding surface. The gold was introduced in twenty-five minutes. The cavity of this bicuspid was very large, and as the distal cavity extended somewhat under the edge of the gum, a little tin foil was inserted in this place, which was followed by Wolrab's gold cylinders. Every layer of gold was examined very carefully by the gentlemen present, and pronounced to be perfectly consolidated.

CLINIC GIVEN THE SAME DAY AT 1.30 P. M. AT THE NEW YORK COLLEGE OF DENTISTRY.

Dr. Herbst made and explained several of the preparations in his cabinet, such as making a plain tooth into a gum tooth, and enameling the face of a plain tooth to obtain another color, fastening regulating plates, and repairing a rubber set with celluloid in a few minutes, all of which have been previously described.

CLINIC GIVEN THURSDAY, JULY 8TH, AT THE ROOMS OF THE S. S. WHITE DENTAL MANUFACTURING COMPANY, CORNER OF NINTH STREET AND BROADWAY, UNDER THE AUSPICES OF THE FIRST DISTRICT DENTAL SOCIETY.

Dr. E. P. Brown was present and filled for Dr. C. F. W. Bödecker the other central incisor, the labial cavity of which in size exactly corresponded to the one filled by Dr. Herbst in the same mouth the day previous. Dr. Brown filled this cavity in twenty-five minutes, using No. 40 gold and the electro-magnetic mallet, which was adjusted in such manner as to give the very hardest blow that can be obtained by this instrument, using an extra battery for the purpose, as Dr. Brown said that he did not wish to be any longer in operating than Dr. Herbst had been on the previous day. After completion, the surfaces of both were entirely smooth and the edges perfect in every respect, but the difference in sensation experienced by the patient during the introduction of the gold was very great. Every person who has experienced both methods of filling will declare a preference for the Herbst method.

Dr. Herbst, at the same clinic, filled for Dr. Chas. Degenhardt the right upper first molar, and the right upper second bicuspid. The cavities in these teeth faced each other, the one in the molar being in the mesial and grinding surfaces, and that in the bicuspid occupying the distal and grinding surfaces. The two cavities were large, and if filled with the mallet, would have occupied at least one and a half or two hours. Dr. Herbst introduced the gold in those two cavities in twenty minutes, to the satisfaction of every one present at the clinic. Every layer of gold was examined for hardness and adaptation to the walls, and when the last layers were in place, even Dr. Brown pronounced the fillings to be exceedingly hard and perfect. About sixty-five persons were present at this clinic.

CLINIC GIVEN JULY 9TH, AT THE OFFICE OF DR. WM. CARR.

There were present Drs. Wm. Carr, J. C. Neisley, Williams, Schreiter, C. E. Francis and C. F. W. Bödecker. Dr. Herbst filled for the same patient who occupied the chair the day previously, the left lower second bicuspid, in the distal and grinding surface. The excavation had been done the previous day. The operator made a matrix of German silver around the bicuspid, as well as the first lower molar on the right side of the mouth. The rubber dam was adjusted and the tooth filled with Wolrab gold cylinders No. 0, using No. 10 foil upon its grinding surface; the time occupied during the introduction was about twenty minutes. The patient thanked Dr. Herbst for his inventions, declaring that the introduction of the gold by this method gave him no pain whatever.

In the evening, Dr. Carr showed Dr. Herbst a filling in the same mouth which he had completed entirely by the Herbst method during the day. It was a left lower first molar tooth, around which Dr. Herbst had made the matrix. All the teeth which had been filled for this patient were divitalized and somewhat sensitive to percussion, but no pain was experienced during the introduction of the gold.

The filling inserted by Dr. Carr was very nicely made, and presented a beautifully finished and smooth appearance.

CLINIC GIVEN JULY 9TH, 1.30 P. M., AT THE NEW YORK COLLEGE OF DENTISTRY.

Dr. Herbst exhibited before the students the making of diamond drills, engine wheel brushes, red gum enamel, appliances for replanting teeth, making of gold nerve broaches and the general application of matrices, all of which have been previously explained.

CLINIC GIVEN FRIDAY, JULY 9TH, AT 3 O'CLOCK, AT THE ROOMS OF THE S. S. WHITE DENTAL MANUFACTURING COMPANY, UNDER THE AUSPICES OF THE FIRST DISTRICT DENTAL SOCIETY.

Dr. Herbst filled for Dr. Tennison, of New York, the right upper first molar and right upper second bicuspid, the cavities facing each other. The defect in the first bicuspid was exceedingly great; so much so that when excavated the lingual portion of the enamel only remained. All the rest of the dentine was gone, thus leaving an exceedingly thin and delicate wall, through which the point of an instrument was visible before the filling was commenced. Dr. Herbst expressed his great satisfaction at the opportunity afforded him, as he thought he would be able to fill this tooth without fracturing the enamel, which showed deep cracks in several places of the lingual as well as the buccal surface. Before the excavation was commenced he placed the usual German silver ring matrix around both teeth. They were then excavated, the rubber dam applied and the gold inserted. At the cervical border of each cavity a little tin foil was placed, both for the protection of the pulps and to prevent secondary decay. The tin was carefully burnished over the pulp, which was very nearly exposed, and then followed by Wolrab's gold cylinders, No. 0. Dr. Herbst filled both cavities at the same time, alternately adding a layer in each cavity until both were nearly filled, when, unfortunately, a little piece of the lingual wall of the right upper first bicuspid gave away, and had to be repaired with gold without smoothing the rugged edges of the broken enamel. When completed, both fillings were pronounced perfect by every one present.

CLINIC GIVEN SATURDAY, JULY 10TH, AT THE OFFICE OF DR. C. F. W. BÖDECKER.

Dr. Herbst filled the right and left upper central incisors, mesial cavities, for a private patient, each occupying about six minutes' time. The matrix used was the ordinary steel spring about eight inches in length, with a piece of shellac fastened at one end. At this clinic there were present Drs. W. H. Atkinson, W. Atkinson, Schreiter and C. F. W. Bödecker.

CLINIC GIVEN JULY 12TH, AT THE OFFICE OF DR. S. H. GUILFORD, WALNUT STREET, PHILADELPHIA.

Dr. Herbst filled for Dr. Tennison, of New York, the right lower first molar, the cavity involving the grinding and buccal surface, the operation requiring eleven minutes for the introduction of the gold. He also described the different methods of making his matrices. At the same time Dr. Bödecker exhibited the former described little inventions of Drs. Herbst, Berggren and Foerberg. Those present at the meeting, were Drs. E. Bedloe, E. T. Darby, F. M. Dixon, F. D. Gardiner, S. H. Guilford, R. Huey, W. F. Litch, D. N. McQuillen, J. W. Noble, C. N. Pierce, E. R. Pettit, C. E. Pike, H. C. Register, H. M. Sheppard, Jas. Truman, J. N. Woodward, J. R. Yorks, of Philadelphia; J. R. Wood, Camden, N. J.; C. R. Jefferies, Wilmington, Del.; S. B. Luckie, Chester, Pa.; and Drs. R. Schreiter and C. F. W. Bödecker.

CLINIC GIVEN JULY 13TH, 9 A. M., AT THE OFFICE OF DR. S. H. GUILFORD, PHILADELPHIA.

Dr. Herbst filled a very large labial surface of a tooth made out of hippopotamus ivory. The filling was about three-quarters of an inch in length and three-eighths of an inch in breadth. The cavity into which the gold was burnished was a perfectly flat and polished surface, with no undercuts, except a little groove which was cut all around the edge of this tooth. The gentlemen present declared it to be an impossibility to fill it in any other manner with which they were acquainted. Dr. Herbst laid about ten Wolrab cylinders upon the surface, and compressed them by means of an engine burnisher and a piece of cotton. When this was partially condensed the first layer was condensed by means of agate points and the dental engine. Layer after layer was added until the contour was completed, when it was finished with corundum and rubber discs. Before it was quite completed, Dr. Herbst, while trying the resistance of the filling, took the whole of it out of the ivory tooth. All the gentlemen present agreed that the anchorage in the ivory had not been sufficient to securely hold the gold. Dr. Herbst then made a little deeper undercut around the edge of the ivory, and filled the same cavity again, occupying about thirty-five minutes' time. The operation was performed in about the same manner as the previous one, but when done the gold could not be dislodged from the ivory, and presented a beautiful and solid appearance. The gentlemen present were Drs. J. W. Noble, F. D. Gardiner, C. N. Pierce, E. T. Darby, S. H. Guilford, R. Schreiter, and C. F. W. Bödecker.

CLINIC GIVEN JULY 13TH, AT THE OFFICE OF DR. W. G. A. BONWILL, LOCUST STREET, PHILADELPHIA, AT 4 P. M.

In the chair was a patient employed by the S. S. White Dental Manufacturing Company, for whom Dr. Herbst filled a right upper central in the distal surface, and a right upper lateral in the mesial surface. After this, Dr. Herbst again explained the different methods of applying shellac matrices combined with steel springs. Dr. Bonwill at this clinic presented a gentleman for whom he had filled a right upper first molar in the mesial and grinding surface, and a right upper second bicuspid in the distal and grinding surface. Both of these fillings were inserted in less than one hour's time. The cavities in the teeth were very large, the contours had been beautifully restored, and the edges were perfect in every respect. The gentlemen present at this clinic were Drs. D. Neale, W. H. Neale, E. C. Kirk, F. M. Dixon, D. N. McQuillen, J. W. Noble, W. G. A. Bonwill, C. A. Kingsbury, J. A. Woodward, Jas. Truman, W. H. Trueman, A. Tees, F. D. Gardiner, C. N. Pierce, E. R. Pettit, S. H. Guilford, C. J. Essig, R. Schreiter, C. F. W. Bödecker.

DINNER TO DR. HERBST.

On the evening of July 13th, the dentists of Philadelphia honored Dr. W. Herbst with a grand dinner at Hotel Bellevue. The gentlemen present at this occasion were Drs. E. Bedloe, W. G. A. Bonwill, E. T. Darby, F. M. Dixon, C. J. Essig, F. D. Gardiner, J. E. Garretson, S. H. Guilford, R. Huey, Louis Jack, C. A. Kingsbury, E. C. Kirk, W. F. Litch, D. N. McQuillen, D. Neall, J. W. Noble, C. N. Pierce, E. R. Pettit, C. E. Pike, H. C. Register, H. M. Sheppard, Ambler Tees, J. D. Thomas, W. H. Trueman, James Truman, J. W. White, J. A. Woodward, J. R. Yorke, C. A. Timme, R. Schreiter, C. F. W. Bödecker, Mr. F. N. Johnson, Oliver Lund and Gideon Sibley.

CLINIC GIVEN JULY 14TH AT THE ROOMS OF THE S. S. WHITE DENTAL MANUFACTURING COMPANY, 315 FULTON ST., BROOKLYN, UNDER THE AUSPICES OF THE BROOKLYN DENTAL SOCIETY.

Dr. Herbst filled for Dr. L. G. Wilder, of Brooklyn, the right upper first bicuspid, the cavity involving the distal and grinding surfaces. It was filled with Wolrab gold cylinders, but in the last layer Dr. Herbst made use of No. 10 Wolrab foil, dividing the sheet into eight strips and rolling it up into rope form. He also filled for the same patient the right upper central in the distal surface. He used the ordinary steel spring matrix in this cavity, on which he fastened a piece of previously softened shellac, which was pressed upon the lingual surface and a little over the cutting edges of the front teeth, to prevent the steel spring from moving up or down. The filling of this cavity only required about four minutes of time, after which he explained various other methods of applying matrices, principally those made of shellac and German silver.

Dr. J. P. Geran, of No. 65 Green Avenue, Brooklyn, who had attended one of the clinics given by Dr. Herbst in New York, introduced a patient with a very large filling in the mesial and grinding surface of the right upper first molar tooth. This filling had been made by Dr. Geran three days before, entirely by the rotation method. It was very beautifully executed, the surface being hard and polished and the edges perfect. Dr. Geran, after exhibiting his patient, expressed his gratitude to Dr. Herbst for his inventions, at the same time mentioning that for the last three days he had filled teeth, using no other but the Herbst method, and that the patients for whom he operated were unwilling to again submit to the process of malleting. The President, Dr. Wm. Johnson, as well as many other members of the Brooklyn Dental Society, expressed sincere thanks and gratitude to Dr. Herbst for his inventions.

CLINIC GIVEN JULY 17TH AT THE OFFICE OF DR. C. F. W. BÖDECKER.

Dr. Herbst filled for Mrs. H., a private patient of Dr. Bödecker, the right lower first bicuspid, the cavity involving the distal and grinding surface. Before excavating he made, as usual, a matrix of German silver in the manner before described, which, after the excavation had been completed and the rubber dam applied, was again put on and the filling commenced. At the cervical wall of this cavity a thin layer of tin was placed. This was followed by Wolrab's gold cylinders compressed in the usual manner. The cavity involved the distal grinding and a part of the buccal cusp of the tooth, and was of very large size. It was filled in one hour and three minutes. The gentlemen present at this meeting were Drs. W. H. Atkinson, W. Atkinson and C. F. W. Bödecker.

CLINIC GIVEN AT THE MEETING OF THE JERSEY STATE DENTAL SOCIETY, HELD AT ASBURY PARK JULY 22D.

Dr. Herbst filled for Dr. F. H. Batterman the left lower lateral, the cavity occupying the mesial surface, and the right lower lateral on the lingual, distal and labial surfaces, with Wolrab's gold cylinders, using a little of No. 10 foil only in the last layer. He then filled for another patient a left upper canine, involving the mesial and cutting edge. The cavity was very large and the pulp nearly exposed.

Previous to excavating the cavity, the German silver matrix was prepared and explained, and as the cervical portion of the cavity extended very far under the gum, and was exceedingly painful to excavation, the obtundent, made of sulphuric acid saturated with cocaine, and this saturated with sulphuric ether, was made use of with success. After excavating the cavity, the dam was applied, the matrix readjusted and the cavity filled. Previous to the first layer of gold a little tin was burnished upon the cervical portion of the cavity, as well as over the pulp chamber, as the cavity was very sensitive to thermal changes. After the layer of tin had been thus introduced it was followed by Wolrab's gold cylinders No. 0, and in the last layers No. 10 foil prepared in the usual way, and with this he contoured the cutting edge of the eye tooth. At the same clinic Dr. W. G. A. Bonwill filled a right upper second bicuspid in the distal and grinding surface, and a right upper first molar in the mesial and grinding surface, for the purpose of giving Dr. Herbst a chance to witness an operation performed by an American operator.

The teeth were filled with Abbey's No. 10 soft gold foil, which was annealed previous to introduction. Dr. Bonwill completed these two very large operations in about an hour. The operations, when completed, showed great skill, and were highly appreciated by every one present, including Dr. Herbst.

(TO BE CONTINUED.)

